# Supplementary material for: The Expression and Function of Metastases Associated Lung Adenocarcinoma Transcript-1 Long Non-Coding RNA in Subchondral Bone and Osteoblasts from Patients with Osteoarthritis
Source: Cells. 2021 Apr 1;10(4):786. doi: 10.3390/cells10040786 (PMC8066176; doi:10.3390/cells10040786)
Supplement: Supplementary file 1 [file cells-10-00786-s001.zip › Table S1 MALAT1 and joint severity.docx]

**Supplementary Table 1. The relationship between MALAT1 bone expression and OA Severity**

|  | **KL-Grade** | | **Joint Space** | | **Osteophytes** | |
| --- | --- | --- | --- | --- | --- | --- |
|  | **KL3** | **KL4** | **>1mm** | **< 1mm** | **Yes** | **No** |
| **Hip + Knee OA Patients** | 0.003 ± 0.002 (n=4) | 0.14 ± 0.09 (n=13) | 0.003 ± 0.002 (n=4) | 0.14 ± 0.09 (n=13) | 0.11 ± 0.09 (n=12) | 0.10 ± 0.08 (n=5) |
| **Hip OA Patients** | 0.0008 ± 0.0007 (n=2) | 0.19 ± 0.17 (n=6) | 0.0008 ± 0.0007 (n=2) | 0.20 ± 0.17 (n=7) | 0.22 ± 0.19 (n=6) | 0.02 ± 0.02 (n=3) |
| **Knee OA Patients** | 0.005 ± 0.004 (n=2) | 0.07 ± 0.07 (n=6) | 0.005 ± 0.004 (n=2) | 0.08 ± 0.07 (n=6) | 0.008 ± 0.005 (n=6) | 0.21 ± 0.21 (n=2) |

Values represent mean MALAT1 relative expression ± SEM. KL grade, joint space and presence of osteophytes was determined by analysis of pre-operative x-ray radiographs.
